# Gender differences in pulmonary arterial hypertension patients with BMPR2 mutation: a meta-analysis

**DOI:** 10.1186/s12931-020-1309-2

**Published:** 2020-02-06

**Authors:** Xiaoyue Ge, Tiantian Zhu, Xinyi Zhang, Ye Liu, Yonglong Wang, Weifang Zhang

**Affiliations:** 1 Department of Pharmacy, The Second Affiliated Hospital of Nanchang University, Nanchang, 330006 China; 2 Teaching and Research Office of Clinical Pharmacology, College of Pharmacy, Xinxiang Medical University, Xinxiang, 453003 China

**Keywords:** BMPR2 mutations, Pulmonary arterial hypertension, Gender, Meta-analysis

## Abstract

**Objective:**

To investigate the differences in the proportions of BMPR2 mutations in familial hereditary pulmonary arterial hypertension (HPAH) and idiopathic pulmonary arterial hypertension (IPAH) between males and females and the relationship between BMPR2 mutation and PAH severity.

**Methods:**

A computer was used to search the electronic Cochrane Library, PubMed/MEDLINE, and EMBASE databases for clinical trials containing information on the relationship between PAH prognosis and BMPR2 mutations through March 2019. After obtaining the data, a meta-analysis was performed using Review Manager Version 5.3 and Stata.

**Results:**

A meta-analysis was performed on 17 clinical trials (2198 total patients: 644 male, 1554 female). The results showed that among patients with HPAH and IPAH, the BMPR2 mutation rate is higher in male than in female patients [male group (224/644, 34.78%), female group (457/1554, 29.41%), OR = 1.30, 95% CI: 1.06~1.60, *P* = 0.01, I^2^ = 10%]. Furthermore, haemodynamic and functional parameters were more severe in IPAH and HPAH patients with BMPR2 mutations than in those without, and those with BMPR2 mutation were diagnosed at a younger age. The risk of death or transplantation was higher in PAH patients with BMPR2 mutations than in those without (OR = 2.51, 95% CI: 1.29~3.57, *P* = 0.003, I^2^ = 24%). Furthermore, the difference was significant only in male patients (OR = 5.58, 95% CI: 2.16~14.39, *P* = 0.0004, I^2^ = 0%) and not in female patients (OR = 1.41, 95% CI: 0.75~2.67, *P* = 0.29, I^2^ = 0%).

**Conclusion:**

Among patients with HPAH and IPAH, men are more likely to have BMPR2 mutations, which may predict more severe PAH indications and prognosis.

## Backgrounds

Pulmonary arterial hypertension (PAH) refers to the first subgroup of pulmonary hypertension (PH) patients, which is defined as a pulmonary arterial wedge pressure (PAWP) ≤ 15 mmHg and an indexed pulmonary vascular resistance (PVRI) greater than 3 Wood units [1]. Although the use of targeted drugs (such as endothelin receptor antagonists, prostacyclin inhibitors, etc.) has increased the survival rate of PAH patients and significant improvements in PAH outcome have been realized in the modern era (median survival 7 years) compared to the National Institutes of Health registry from the early 1980s (median survival 2.8 years) [2], its long-term prognosis remains poor. The aetiology of PAH involves environmental and genetic factors, and its pathogenesis is complex and has not been fully elucidated to date..

Bone morphogenetic protein (BMP), a multifunctional protein, was originally identified as an osteoinductive component in extracts derived from bone. BMPs play important roles through BMP receptors (BMPRs) in a multitude of processes during embryonic development and adult homeostasis. BMPRs are serine/threonine kinase receptors composing an intracellular serine/threonine kinase domain, a single transmembrane domain, and a short extracellular domain containing 10–12 cysteine residues. The BMP type II receptor (BMPRII), activin type II receptor (ActRII), and activin type IIB receptor (ActRIIB) are three type II BMPRs present in mammals. Autosomal dominant mutation causing haploinsufficiency or loss of function of BMPR2 is the most common cause of PAH. Studies have reported more than 298 BMPR2 mutations are responsible for 55 to 70% of heritable PAH (HPAH) and 11 to 40% of idiopathic PAH (IPAH) [3]. What’s more, the BMPR2 signaling pathway is also impaired in PAH patients without BMPR2 mutations [4]. Despite the impact of BMPR2 as the main genetic factor for PAH, currently, our understanding of the specific mechanisms of BMPR2 in PAH is incomplete. Unravelling these uncertainties may be could explain why only 20% of BMPR2 mutation carriers develop advanced PAH [5] and might predict which mutation carriers will further develop PAH. It is now recommended by the European guidelines for the management of PAH that patients recently diagnosed with idiopathic, heritable, or anorexigen-associated PAH should be offered genetic counselling and screening for BMPR2 mutations, mainly to enable predictive genetic testing of relatives. Studies have suggested that patients with PAH who carry causal BMPR2 mutations may present at an earlier age and have more severe haemodynamic compromise [6]. Additionally, there is a “sex paradox” exists in PAH while women are more likely than men to develop PAH, men have worse outcomes than those observed in their opposite sex counterparts [7]. Therefore, among PAH patients, the question of whether there is a gender difference in BMPR2 mutation is still unclear. In this article, we sought to combine recent clinical research investigating BMPR2 and PAH to present an evidence-based meta-analysis and explain the scientific phenomenon of gender differences in the development of PAH disease. Moreover, these data provide clinical guidance for the treatment of gender-based differences in PAH.

## Methods

### Literature search

We obtained individual participant data from studies identified through systematic searches of the published literature performed using the Cochrane Library, PubMed/MEDLINE, and EMBASE databases (the following search terms were used: “BMPR2 or bone morphogenetic protein receptor type 2” and “pulmonary hypertension”) up to March 2019. The electronic searches were specifically performed to obtain articles in peer-reviewed journals. Additional data not identified in the electronic databases, especially original data that were absent from published articles, were collected from other data resources. We also performed an additional search of the references of the retrieved studies. Specifically, we contacted the corresponding authors to obtain original data that were not reported in the identified published articles.

### Selection of studies for inclusion in the review

Cohort studies were included if they met the following criteria: 1) Type of study: retrospective or prospective; 2) Types of participants: idiopathic PAH or heritable PAH with a date of PAH diagnosis defined as the date of diagnostic right heart catheterization; 3) Type of interventions: patients underwent sequencing for BMPR2 mutations; and 4) Type of outcome measure: the composite of death or lung transplantation. The exclusion criteria were as follows: 1) Duplicate reports describing the same cohort; 2) Certain publication types, such as conference abstracts, letters, comments, case reports and editorials; and 3) Studies not published in English.

### Data extraction and quality evaluation

All studies retrieved by the search strategy were independently screened by 2 reviewers (XYG and TTZ). The initial prescreening was performed by reading the titles and abstracts to select relevant studies for further data extraction. Secondary selection was conducted by comprehensively reviewing the full text of all initially identified articles to determine whether the necessary information was reported. Disagreements were resolved through discussion or consultation with the 3rd reviewer (YL). Data were extracted according to predetermined criteria, and a quality evaluation of the individual studies was then performed in accordance with the methodological standards proposed by McGinn and colleagues.

### Appraisal of the risk of bias of the included studies

Two reviewers (XYG and TTZ) independently evaluated the risk of bias in accordance with the Cochrane Collaboration’s “risk of publication bias” tool. Disagreements were resolved by consensus. Potential publication bias was evaluated by visually inspecting funnel plots and by analytical appraisal based on the Begg’s adjusted-rank correlation test and Egger’s linear regression test. According to the Begg or Egger methods for evaluating publication bias, a two-sided *P* value of 0.10 or less was regarded as significant.

### Statistical analysis

Statistical analyses were performed using Review Manager Version 5.3, Stata and SPSS Statistics. The chi-square statistic and independent-samples T tests were used to assess differences in the baseline characteristics of the two groups. The odds ratio (OR) was calculated and presented with the 95% confidence interval (CI) for summary estimates. Due to the heterogeneity among the included studies, appropriate statistical models were selected to ensure that the statistical data were estimated correctly. Cochran’s chi-square test was performed, and the I^2^ statistic was determined to evaluate the heterogeneity among the included studies. Cochran’s chi-square test was used to determine whether the observed difference may be due to chance alone. A low *P* value (cut-off of 0.10) was taken to indicate the presence of significant heterogeneity among different studies. The I^2^ statistic describes the percentage of total variation across studies due to significant heterogeneity rather than random chance. An I^2^ statistic greater than 75% suggests considerable heterogeneity among the studies. Statistical significance was set at a *P* value < 0.05.

## Results

### The review process and characteristics of the included studies

**Fig. 1:**
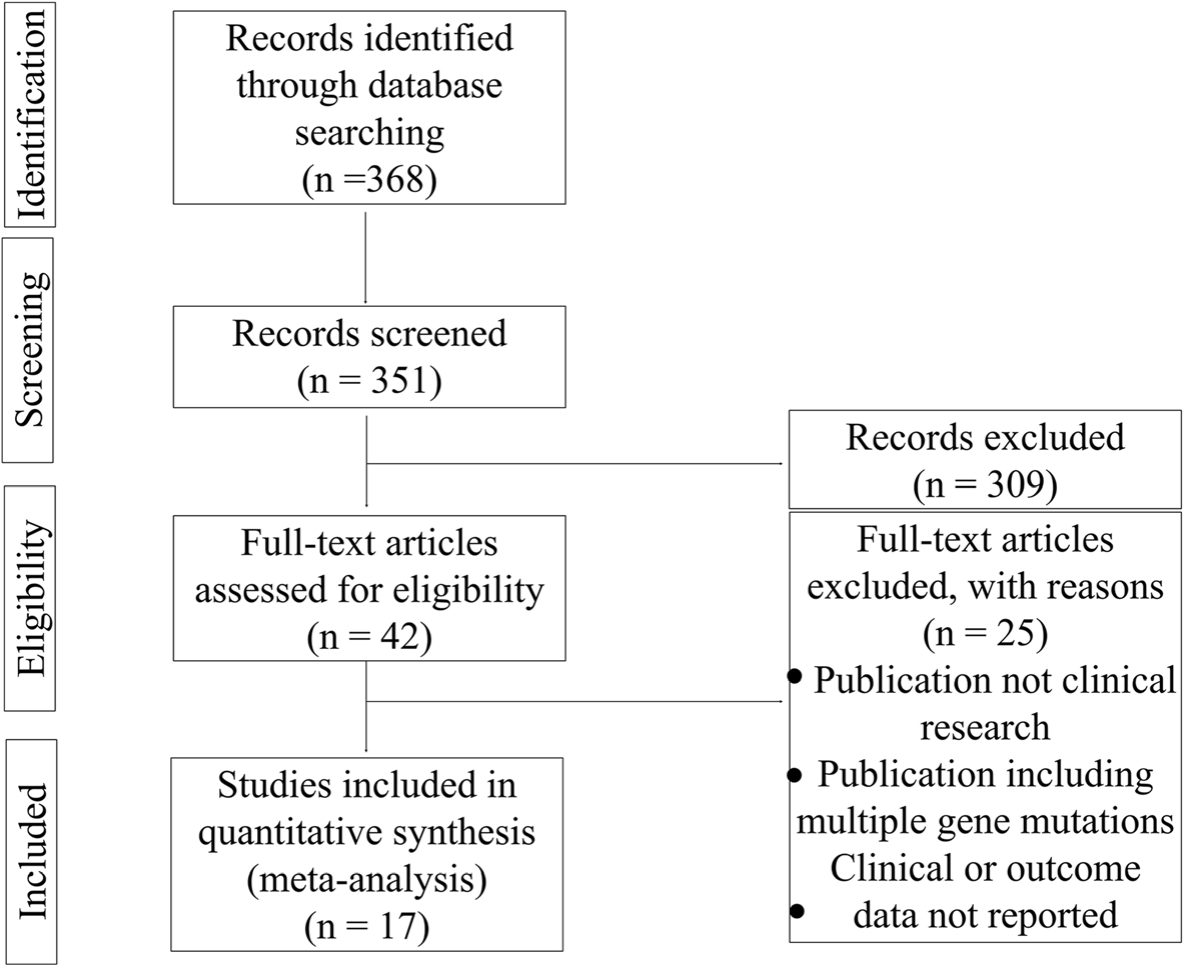
Study and patient selection

Figure 1 shows the flowchart for the inclusion and exclusion of studies and patients. Literature search results and characteristics were initially obtained from 368 articles published in English, and 351 articles were obtained as full text. A total of 309 papers were removed after reviewing titles and abstracts were reviewed. Then, the full text of each of the remaining 42 articles was retrieved for further review to determine whether they met the predetermined criteria. Finally, 17 papers were identified and included in the present study [8–24].

The general characteristics of the included studies were shown in Table 1. The publication year ranged from 2006 to 2018. Based on the data in the 17 eligible trials, a total of 2198 PAH patients were enrolled; among these, 681 (30.98%) were BMPR2 mutation carriers and 1517 (69.02%) were noncarriers. The sex ratio of female to male patients was 2.41 (*n* = 1554/644) in the total population, 2.04 (*n* = 457/224) among BMPR2 mutation carriers, and 2.61:1 (*n* = 1097/420) among noncarriers. IPAH and HPAH patients with BMPR2 mutations had more severe haemodynamic and functional parameters than those observed in noncarriers, and those with BMPR2 mutation were diagnosed at a younger age. (Table 2).

**Table 1:** General characteristics of the included studies

Study ID	PH types for data extraction	Area	Participants (N)	mutation /non-mutation (N/N)	Male/Female (N/N)	Outcomes	Period
Austin 2009 [8]	HPAH, IPAH	US	147	106/41	43/104	death or lung transplantation	up to date as of 2009.03
Bruggen 2016 [12]	HPAH, IPAH	Netherlands	95	28/67	23/72	death or heart/lung transplantaton	1995.03 to 2014.10
Chida 2012 [20]	IPAH, HPAH	Japan and China	54	18/36	24/30	death	1995.01.01 to 2011.03.31
Elliott 2006 [21]	HPAH, IPAH	US	67	27/40	14/53	NA	1994.07 -
Gamou 2017 [14]	HPAH	Japan	117	39/78	31/86	NA	2015
Ghigna 2016 [10]	HPAH, IPAH	French	44	23/21	16/28	lung transplantation	2005–2014
Girerd 2010 [9]	HPAH, IPAH	French	382	115/267	113/269	death or lung transplantation	2004.01.01–2010.04.01
Isobe 2016 [18]	HPAH, IPAH	Japan	59	23/36	17/42	death	2000.08–2015.10
Kabata 2013 [13]	HPAH, IPAH	Japan	49	18/31	17/32	death or lung transplantation	1999.10–2007.03
Liu 2012 [11]	HPAH, IPAH	China	305	50/255	87/218	death	2006.01.01–2010.08.31
Mutlu 2016 [23]	IPAH, CHD-PAH	Turkish	43	1/42	21/22	NA	2011–2012
Navas 2016 [19]	HPAH, IPAH	Spain	165	24/141	41/124	death or lung transplantation	2011.01–2015.05.01
Pfarr 2011 [17]	HPAH, IPAH	Germany	228	49/179	62/166	NA	2006.01–2009.12
Pousada 2014 [22]	IPAH and Associated PAH	Spain	41	9/32	21/20	NA	14 months
Rosenzweig 2008 [24]	HPAH, IPAH	US	147	23/124	49/98	NA	1991–2005
Sztrymf 2008 [15]	HPAH, IPAH	French	233	68/165	66/167	death or lung transplantation	2004.01–2007.06
Yang 2018 [16]	HPAH, IPAH	China	185	56/129	48/137	NA	2016–2017

*NA* not available, *HAPH* heritable PAH, *IPAH* idiopathic PAH

**Table 2:** Hemodynamic and cardiac functional parameters according to the BMPR2 mutations of pulmonary hypertension

Variables	Mutations	Non-mutations	MD/OR(95%CI)	*P* value
Age at diagnosis (yrs)	35.12 ± 12.79 (556)	40.17 ± 16.25 (1174)	−3.70 [−6.52, −0.87]	<0.00001
6MWD (m)	363.12 ± 113.97 (332)	369.22 ± 116.82 (863)	−14.81 [−45.24, 15.62]	0.34
mPAP (mmHg)	61.21 ± 12.73 (570)	56.37 ± 14.08 (1197)	4.82 [2.38, 7.25]	0.0001
PVR (Wood units)	18.65 ± 8.69 (557)	15.11 ± 7.8 (1166)	3.90 [3.23, 4.57]	<0.000
CI (L/ml/m^2^)	2.08 ± 0.65 (547)	2.51 ± 0.86 (1156)	−0.44 [−0.63, − 0.24]	<0.000
RVP (mmHg)	8.30 ± 4.99 (405)	7.41 ± 5.60, 835 (835)	−0.32 [− 0.91, 0.27]	0.29
NYHA III	67.95% (312)	70.22% (648)	0.84 [0.62, 1.14]	0.26
NYHA IV	11.54% (312)	6.94% (648)	1.71 [1.07, 2.72]	0.02

*6MWD* 6 min walk distance, *mPAP* mean PAP, *PVR* pulmonary vascular resistance, *CI* cardiac index, *RVP* right ventricular pressure; these results are expressed as mean ± SD. *NYHA* new york heart association functional classification, expressed as % (patients number); *MD* mean difference, *OR* odd ratio, *95% CI* 95% confidence interval

### Quality evaluation

Table 3 showed that the included studies generally included patients with a variety of disease severities and who were generally selected in an unbiased fashion. In addition, the follow-up durations of the patients and issues relating to blinding were also acceptable. Therefore, both external and internal validity were adequate.

**Table 3:** Quality Evaluation of the Individual Studies

Study ID	Q1	Q2	Q3	Q4	Q5
Austin 2009 [8]	Yes	No	Yes	Yes	Yes
Bruggen 2016 [12]	Yes	No	Yes	Yes	Yes
Chida 2012 [20]	Yes	No	Yes	No	No
Elliott 2006 [21]	Yes	No	Yes	Yes	Yes
Gamou 2017 [14]	Yes	No	Yes	Yes	Yes
Ghigna 2016 [10]	Yes	No	Yes	Yes	Yes
Girerd 2010 [9]	Yes	No	Yes	Yes	Yes
Isobe 2016 [18]	Yes	No	Yes	Yes	Yes
Kabata 2013 [13]	Yes	No	Yes	Yes	Yes
Liu 2012 [11]	Yes	No	Yes	Yes	Yes
Mutlu 2016 [23]	Yes	No	Yes	Yes	Yes
Navas 2016 [19]	Yes	No	Yes	Yes	Yes
Pfarr 2011 [17]	Yes	No	Yes	Yes	Yes
Pousada 2014 [22]	Yes	No	Yes	Yes	Yes
Rosenzweig 2008 [24]	Yes	No	Yes	Yes	Yes
Sztrymf 2008 [15]	Yes	No	Yes	Yes	Yes
Yang 2018 [16]	Yes	No	Yes	Yes	Yes

*Q* question; Q1 (external validity): Did the patients represent a variety of disease severities?; Q2 (external validity): Did the included study exhibit bias?; Q3 (internal validity): Was the follow-up percentage of all enrolled patients greater than 80%?; Q4 (internal validity): Were the predictors to be evaluated blinded to the outcome events?; Q5 (internal validity): Were the outcome events blinded to the predictors?

### Bias assessment and consistency test

**Fig. 2:**
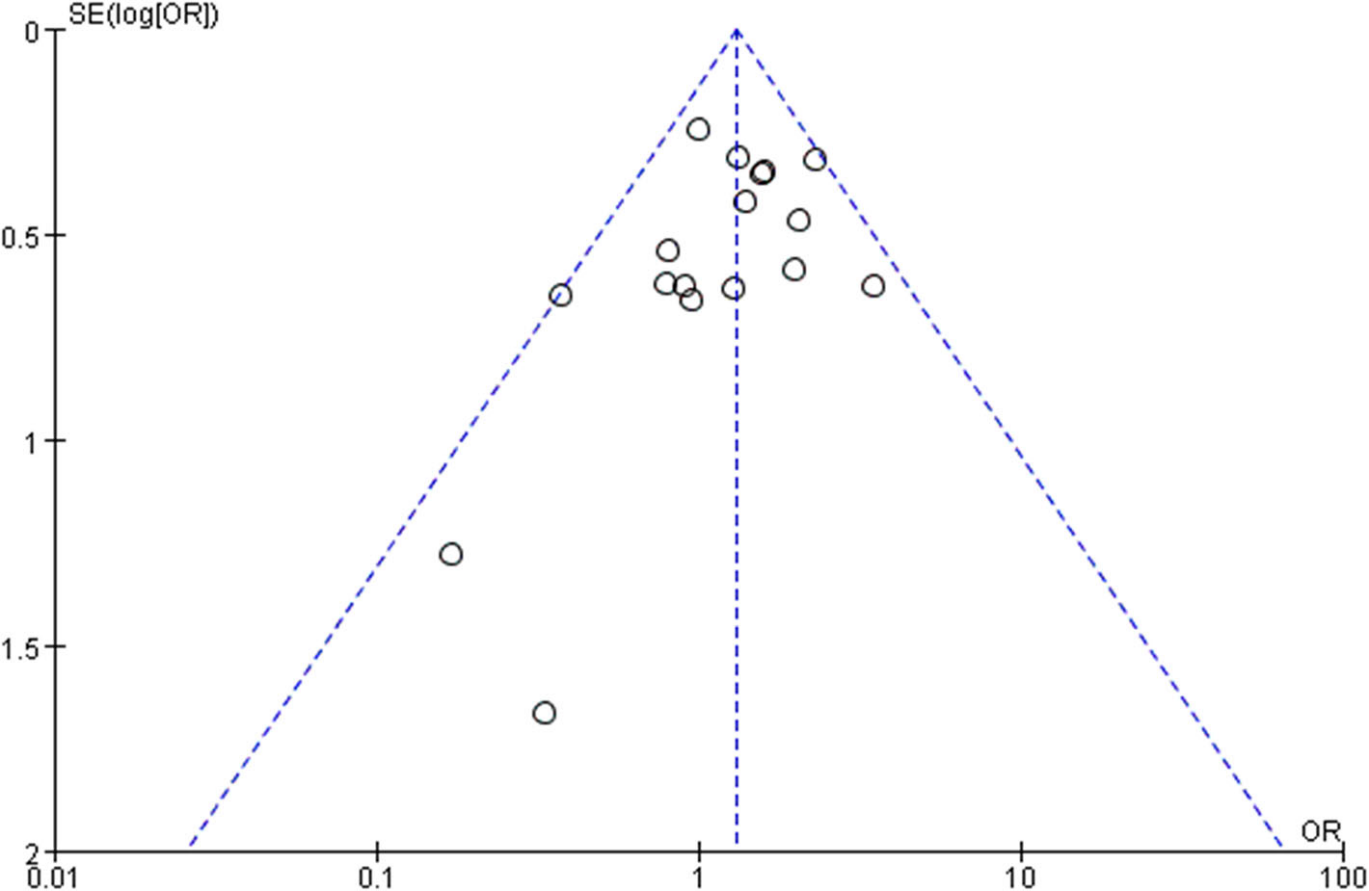
Funnel plot showing all studies included in the bias analysis. logor, logarithm of odds ratio; s.e.: standard error

Visual inspection of a funnel plot (Fig. 2) showed that it had a symmetrical shape, and quantitative evaluation suggested there was no significant publication bias (Egger’s test [*P* = 0.249], Begg’s test [*P* = 0.232]).

### BMPR2 mutations in male and female patients with IPAH or HPAH

**Fig. 3:**
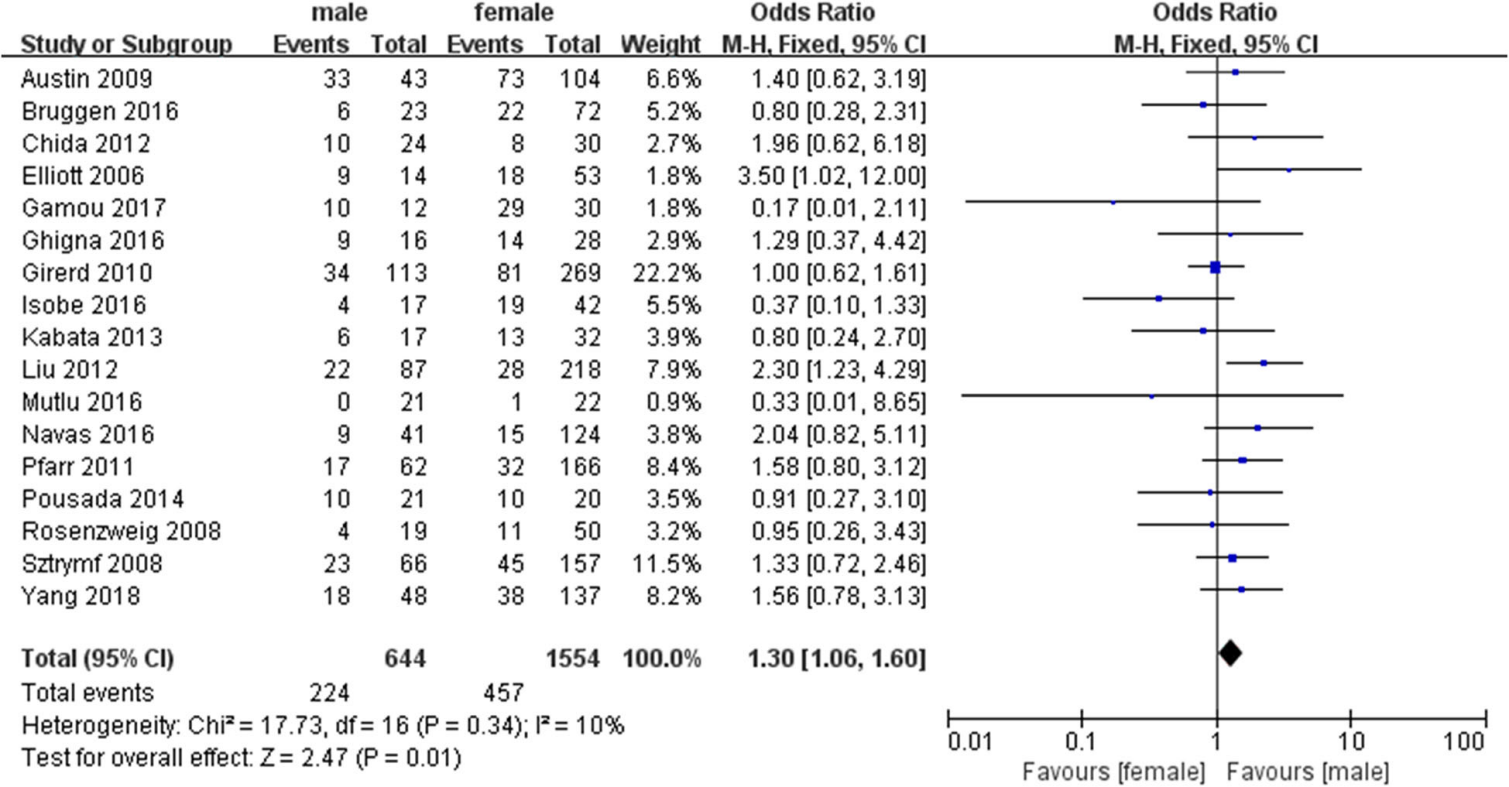
Forest map comparing BMPR2 mutations between male and female PAH patients. *P* < 0.05 was considered statistically significant

In a pooled analysis of all included patients with BMPR2 mutations, we observed very low heterogeneity (I^2^: 10%), indicating that the variability among these studies was acceptable. Among all included PAH patients, 644 patients were male, including 224 patients (34.78%) with BMPR2 mutations. Among the 1554 female patients, only 457 patients (29.41%) had BMPR2 mutations. When the data were pooled across these studies, the results (Fig. 3) showed that the male patients were significantly more likely than the female patients to have BMPR2 mutations (OR = 1.30, 95% CI: 1.06~1.60, *P* = 0.01).

### Mortality or lung transplantation rate

**Fig. 4:**
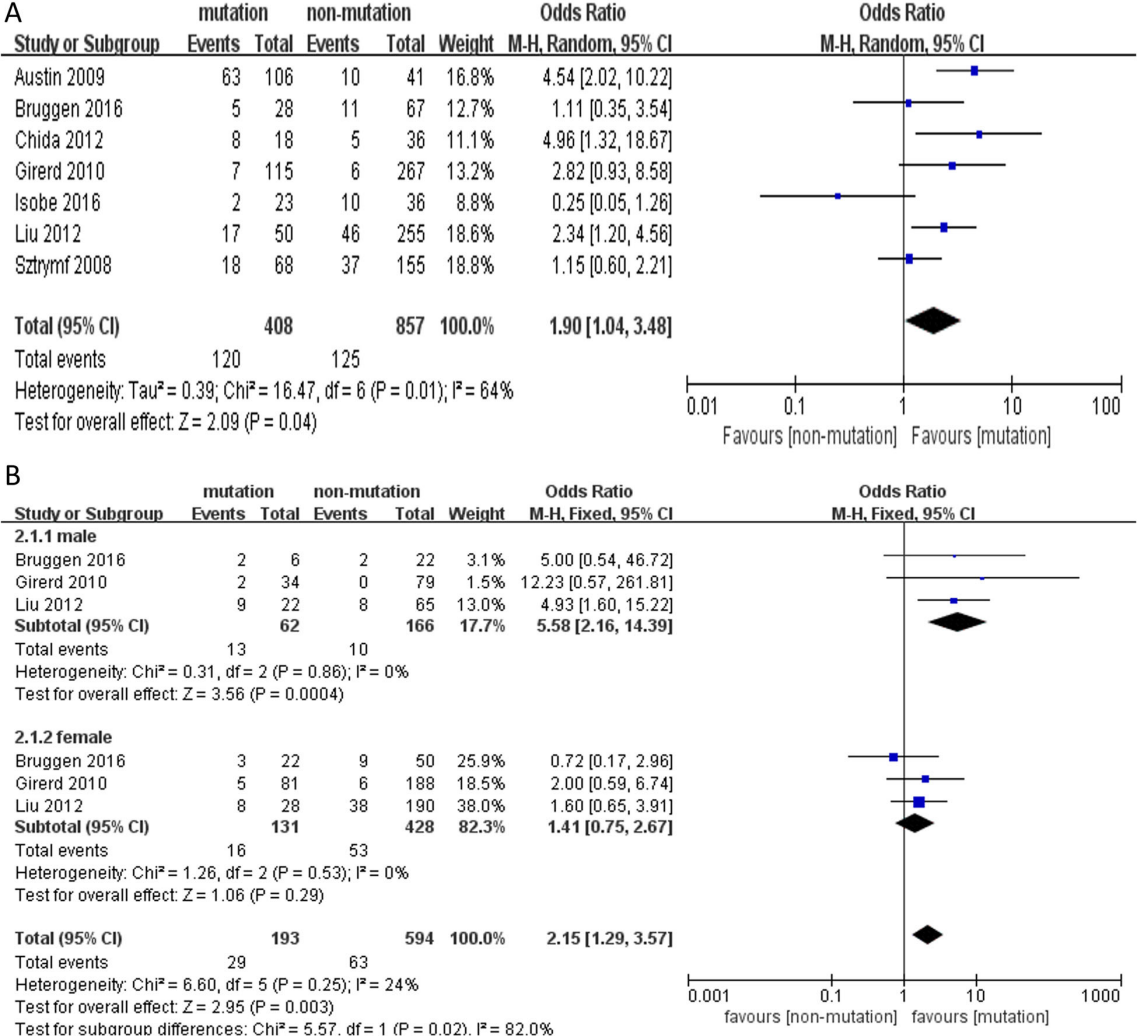
Forest map comparing death or lung transplantation between PAH patients with and without BMPR2 mutations. **a** death or lung transplantation in different PAH patients, **b** subgroup analyses for death or lung transplantation by different gender. *P* < 0.05 was considered statistically significant

Compared to non-carriers of BMPR2 mutation, carriers were associated with significantly higher mortality and lung transplantation rates (OR = 1.90, 95% CI: 1.04~3.48, *P* = 0.04, I^2^ = 64%) (Fig. 4a). Furthermore, we performed subgroup analyses among the studies according to gender to further explain the high heterogeneity observed among the trials. Similarly, the risk of death or transplantation was higher in PAH patients with BMPR2 mutations than in those without (OR = 2.15, 95% CI: 1.29~3.57, *P* = 0.003, I^2^ = 24%). Moreover, this difference was significant only in male patients (OR = 5.58, 95% CI: 2.16~14.39, *P* = 0.0004, I^2^ = 0%) and not in female patients (OR = 1.41, 95% CI: 0.75~2.67, *P* = 0.29, I^2^ = 0%) (Fig. 4b).

## Discussion

Of all BMPRs, BMPR2 is the most relevant to PAH. BMPR2 mutations were the first BMPR mutations to be discovered, and they are the most extensively studied mutations of those known to underlie HPAH. Transgenic mice with PAH expressing a dominant-negative BMPRII gene display aberrant pulmonary vascular cell phenotypes, including apoptosis of endothelial cells (ECs) and excessive proliferation of medial smooth muscle cells (SMCs) [25]. Metabolomic analysis demonstrated that BMPR2 mutations were associated with a wide range of metabolic abnormalities, including oxidative injury and insulin resistance in human pulmonary ECs. Furthermore, BMPR2 deficiency aggravates endothelial inflammatory responses, thereby contributing to adverse vascular remodeling [26]. Homozygous BMPR2(−/−) knockout mouse died in utero whereas heterozygous BMPR2(+/−) mice were viable but did not develop PAH spontaneously, even in the presence of a second hit such as hypoxia, mouse did not develop severe forms of PAH [27]. Given that BMP ligands and their receptors play important roles in disease progression, the regulation of this signal could function as a therapeutic target.

As mentioned earlier, there is a “sex paradox” in PH that it has long been known females have a higher susceptibility than males to PAH, in which the most recent figures show that the female-to-male ratio is 4:1 [28]. In a large cohort of individuals with BMPR2 receptor mutations (including those with IPAH or HPAH or drug- and toxin-induced PAH), approximately 70% of the patients were women [6]. Similar to previous studies, in our study, 457 of 681 PAH patients with BMPR2 mutations were women. Interestingly, among all female PAH patients, the proportion who had BMPR2 mutations was lower than the proportion of male PH patients with BMPR2 mutations out of all male PAH patients. Therefore, our and previous studies suggest that the pathogenesis of PAH may be more complicated in female than male PAH patients, and that the influence of BMPR2 mutations may be modified by additional unknown factors in female patients. A large and growing body of literatures have investigated the cross-talk between BMPR2 and oestrogen signaling, which has been proposed as a critical mechanistic driver responsible for the female predominance of PAH. Austin ED ect. Showed that when oestrogen receptor alpha binds to the BMPR2 promoter, BMPR2 gene expression was reduced in females [29]. Mair and colleagues examined the expression of aromatase (a member of the cytochrome P-450 superfamily that synthesizes oestrogens via the aromatization of androgens) in human pulmonary artery smooth muscle cells (hPASMCs) and demonstrated that the level of aromatase was 12-fold higher in cells derived from postmenopausal women than in PASMCs derived from similarly aged men [30]. Furthermore, a large number of recent studies have focused on differences in hormone levels but have ignored the most fundamental difference between males and females: the sex chromosomes (XX versus XY). Yan and colleagues demonstrated that SRY on the Y chromosome binds to and positively regulates the BMPR2 promoter to reduce the prevalence of PAH in males [31]. Moreover, studies have demonstrated mitochondria also play an important role in PAH [32]. Does female mitochondria also be differ from male mitochondria? It’s true! Mitochondria are passed to the offspring only from mother. Female mitochondria can respond to evolutionary pressure because they are passed on to the offspring. However, male mitochondria are not responsive to such pressures because they are locked in the host cell and cannot be passed on [33].

Studies have increasingly shown that inflammation plays an important role in PAH and the inflammatory insult is also considered as a second hit potential triggering the pathogenesis of PAH [34]. The occurrence of PAH is related to various inflammatory factors, such as systemic inflammatory response, human immunodeficiency virus infection, autoimmune disease, etc. Evidence suggesting that inflammation is involved in the development of PAH was first presented in 1994. Tuder found that macrophages, T cells, B cells and other inflammatory cells infiltrate surrounding injured vessels in PAH patients with pulmonary plexiform lesions [35], and many studies have found significantly increased levels of inflammatory markers in the blood of PAH patients, including C-reactive protein (CRP), interleukins (IL, such as IL-1, IL-6, IL-8, and IL-10), monocyte chemotactic protein 1 (MCP-1), tumor necrosis factor-α (TNF-α), and high mobility group box chromosomal protein 1 (HMGB1) [36]. Furthermore, differences in the immune system have been identified between men and women. Therefore, we predict that these sex-related differences in the immune system contribute to the PH “sex paradox”. In addition to IPAH and HPAH, other World Health Organization (WHO) Group 1 PAH subgroups are also characterized by a female predominance [7], including connective tissue disease (CTD) and portopulmonary hypertension-associated PAH. CTD is more common in women than men, and when associated with PAH, female/male ratios between 3.8:1 and 10:1 have been reported [10]. In systemic sclerosis, women are eight times more likely than men to be affected by PAH, and in systemic lupus erythematosus, women are 17 times more likely than men to be affected by PAH [8].

Our meta-analysis further confirmed a positive correlation between BMPR2 mutations and the severity of PAH, which is consistent with previous studies [9], reporting that PAH patients carrying BMPR2 mutations have a higher mean pulmonary artery pressure and a lower cardiac index. Similar to the Evans study, we also found that in patients with PAH, the risk of death or transplantation was higher among those with BMPR2 mutations than those without [6]. However, it is worth noting that when we conducted subgroup analyses according to gender to explore the sources of heterogeneity, we found that the difference was significant only in male patients but not in female patients. To explain this finding, we first focused on differences in sex hormones between males and females. Similar to the “sex paradox”, there are also “oestrogen paradoxes” in PAH [7]. At low doses, oestrogen acts as a pro-oxidant, whereas at higher doses, it acts to suppress oxidative stress. In addition to its dose-effect, studies have demonstrated that some oestrogen metabolites (such as 17-β oestradiol and 2-OH-estradiol) can also promote antiproliferative and proapoptotic signals, inhibit oxidative stress and collagen deposition, and protect right ventricular function in PAH, while other metabolites (such as 16-α-OH-oestradiol) have the opposite effects [28]. The effects of key enzymes in the lungs (such as CYP1B1 and catechol-O-methyl-transferase (COMT)) have also been explored. CYP1B1 is the most efficient oestradiol hydroxylase, and the 2-OH-estradiol/16α-OH-estrone ratio is an indicator of CYP1B1 activity [37]. In the presence of COMT, 2-Methoxy-estradiol is rapidly formed. The imbalance of oestrogen metabolites has been proposed to be the basis of the differential effects of oestrogens on male versus female pulmonary vascular cells [38]. As mentioned earlier, in addition to hormonal factors, there are also differences in the immune system between men and women. Some studies have demonstrated that Tregs are more critical for the maintenance of immune homeostasis in females than in males. Treg cells can maintain immune homeostasis and suppress inflammatory responses by regulating the function of effector T cells. Under physiological conditions, Th17 and Treg are in a dynamic equilibrium. In PAH, the balance of Th17 and Treg is broken, and Th17 cells, which have pro-inflammatory effects, are increased, and the activation of Treg cells, which exert anti-inflammatory protective effects, is reduced [39]. Osman and colleagues found that Tregs suppressed inflammation, immune dysregulation and vascular remodeling in females and that they thereby exert a protective effect in PAH models [33]. Moreover, metabolic theory suggests that vascular cell mitochondria can induce a proliferative, antiapoptotic phenotype and the inflammasome, thus initiating a cascade of events that increases the levels of many inflammatory cytokines described in PAH [40]. Hence, female mitochondria may be different from male mitochondria. Oocytes cell mitochondria have different ultrastructures, slower metabolic activity and lower levels of ROS production than those observed in sperm cell mitochondria. Importantly, mitochondria can strongly regulate the functions of T cells. For example, mitochondrial suppression of oxidative phosphorylation is a necessary step preceding T cell activation [41].

Our results also indicate that screening for BMPRR2 mutations is more important in men than in women. First, we presented evidence from 17 published clinical trials in this review and suggested that among 2198 PAH patients, BMPR2 mutations accounted for a higher proportion of all factors leading to PAH in men than women. Second, the PAH patients with BMPR2 mutations had more severe haemodynamic and functional parameters than noncarriers, and the carriers were diagnosed at younger ages. The risk of death or transplantation in PAH patients with BMPR2 mutations was higher than that in those without such mutations. The third and last, the difference was significant only in male patients. Therefore, treatment of aberrant BMPR2 expression may exert better effects in male IPAH and HPAH patients than in female patients. This study also has some shortcomings. We found that male PAH patients are more likely than female patients to have BMPR2 mutations, but whether this difference is related to the severity of male PAH has not been further explored. The main reason is that many end points of the included clinical studies were only systematically compared with regard to the presence or absence of BMPR2 mutations, and no stratified analysis of males and females has been performed. We recommend a previously completed or ongoing trial or registration of PAH should be analyzed in a gender subgroup analysis of patients with BMPR2 mutations to determine whether gender differences in BMPR2 mutations are associated with gender differences in disease severity among this patient population. Moreover, some heterogeneity was observed in the mortality or lung transplantation rates among the trials. In Isobe’s study, we observed that the overall results were consistent with other studies. The all-cause mortality was similar between the BMPR2 carriers and non-carriers, but in contrast to the other included studies, this study found that the all-cause mortality was higher among the BMPR2 non-carriers. The reasons may be summarized as follows: the population explored in this study were treated with PGI_2_, while the other studies did not involve treatment, and 13.6% of the entire cohort in this study had familial pulmonary hypertension, which is twice the 6% reported in the U.S. registry conducted in the 1980s. Thus, we cannot deny the possibility that ethnicity contributes to the impact of BMPR2 mutations on the outcome. Indeed, the heterogeneity was greatly reduced after eliminating Isobe’s study (from 64 to 49%, data not shown). Thus, a worldwide, large-scaled prospective study should be performed in the near future to elucidate the differences in among response to the currently available combination therapies between for BMPR2. What’s more, there are studies have reported that gender-based differences in PAH prevalence appear to be diminished among older patients [42]. Therefore, an another important modifier of the relationship between gender and outcomes in PAH may be is age, which suggests that temporal changes in the hormonal milieu may impact disease risk and severity throughout the lifespan [43]. Just as we discussed before, race/ethnicity may also modify the relationship between sex and PAH. So, we probably group it more finely, such as by region, age, etc., to further explain the high heterogeneity.

## Conclusion

In this review, we presented evidence from 17 published clinical trials and suggested that among 2198 HPAH and IPAH patients, BMPR2 mutations accounted for a higher proportion out of all factors that led to PAH in men than in women. Moreover, IPAH and HPAH patients with BMPR2 mutations had more severe haemodynamic and functional parameters than noncarriers, and those carriers were diagnosed at younger ages. The higher risk of death or transplantation was higher in PAH patients with BMPR2 mutations than in those without. Furthermore, the difference was significant only in male patients. Therefore, the restoration of BMPR2 expression and regulation of the BMPR2 signalling pathway are expected to represent an effective method for the treatment of PAH, and given the findings of our meta-analysis results, we speculate that the therapeutic effects of restoring BMPR2 expression may be better in male than that in female PAH patients.

## Data Availability

Data are available from the authors upon request.

## Abbreviations

BMP: Bone morphogenetic protein
BMPRs: BMP receptors
CI: Confidence interval
CTD: Connective tissue disease
ECs: Endothelial cells
HPAH: Hereditary pulmonary arterial hypertension
hPASMCs: Human pulmonary artery smooth muscle cells.
IPAH: Idiopathic pulmonary arterial hypertension
mPAP: Mean pulmonary artery pressure
OR: Odds ratio
PAH: Pulmonary arterial hypertension
PH: Pulmonary hypertension
SMCs: Smooth muscle cells
WHO: World Health Organization
